# Machine learning models for reinjury risk prediction using cardiopulmonary exercise testing (CPET) data: optimizing athlete recovery

**DOI:** 10.1186/s13040-025-00431-2

**Published:** 2025-02-17

**Authors:** Arezoo Abasi, Ahmad Nazari, Azar Moezy, Seyed Ali Fatemi Aghda

**Affiliations:** 1 Department of Health Information Management, School of Health Management and Information Sciences, Iran University of Medical Sciences, Tehran, Iran; 2 Student Research and Technology Committee, School of Health Management and Information Sciences, Iran University of Medical Sciences, Tehran, Iran; 3 Department of Sports and Exercise Medicine, School of Medicine, Iran University of Medical Sciences, Tehran, Iran; 4 Fakher Mechatronic Research Center, Kerman University of Medical Sciences, Kerman, Iran; 5 Research Center for Health Technology Assessment and Medical Informatics, School of Public Health, Shahid Sadoughi University of Medical Sciences, Yazd, Iran

**Keywords:** Athlete recovery, Cardiopulmonary exercise testing, CPET, Injury prevention strategies, Machine learning, Reinjury risk prediction, Sports medicine

## Abstract

**Background:**

Cardiopulmonary Exercise Testing (CPET) provides detailed insights into athletes’ cardiovascular and pulmonary function, making it a valuable tool in assessing recovery and injury risks. However, traditional statistical models often fail to leverage the full potential of CPET data in predicting reinjury. Machine learning (ML) algorithms offer promising capabilities in uncovering complex patterns within this data, allowing for more accurate injury risk assessment.

**Objective:**

This study aimed to develop machine learning models to predict reinjury risk among elite soccer players using CPET data. Specifically, we sought to identify key physiological and performance variables that correlate with reinjury and to evaluate the performance of various ML algorithms in generating accurate predictions.

**Methods:**

A dataset of 256 elite soccer players from 16 national and top-tier teams in Iran was analyzed, incorporating physiological variables and categorical data. Several machine learning models, including CatBoost, SVM, Random Forest, and XGBoost, were employed to predict reinjury risk. Model performance was assessed using metrics such as accuracy, precision, recall, F1-score, AUC, and SHAP values to ensure robust evaluation and interpretability.

**Results:**

CatBoost and SVM exhibited the best performance, with CatBoost achieving the highest accuracy (0.9138) and F1-score (0.9148), and SVM achieving the highest AUC (0.9725). A significant association was found between a history of concussion and reinjury risk (χ² = 13.0360, *p* = 0.0015), highlighting the importance of neurological recovery in preventing future injuries. Heart rate metrics, particularly HRmax and HR2, were also significantly lower in players who experienced reinjury, indicating reduced cardiovascular capacity in this group.

**Conclusion:**

Machine learning models, particularly CatBoost and SVM, provide promising tools for predicting reinjury risk using CPET data. These models offer clinicians more precise, data-driven insights into athlete recovery and risk management. Future research should explore the integration of external factors such as training load and psychological readiness to further refine these predictions and enhance injury prevention protocols.

## Introduction

Cardiopulmonary Exercise Testing (CPET) has become an essential modality in evaluating exercise physiology, providing substantial information about cardiac and pulmonary physiology [1, 2]. By measuring key parameters such as oxygen consumption (VO_2_), carbon dioxide production (VCO_2_), heart rate, and ventilation, CPET paints a comprehensive picture of athletes’ cardiorespiratory training [3, 4]. Applications of CPET as a prognostic and predictive tool has gained increasing attention in recent years [5]. Once primarily used to assess cardiorespiratory fitness, CPET is now a critical component in various clinical fields, from evaluating exercise tolerance in individuals with chronic illnesses to conducting preoperative assessments in high-risk patients and in the athletic performance assessment [6–8]. 

In the broader sports analytics field, machine learning has demonstrated success in forecasting performance and understanding injury patterns. For instance, research in basketball analytics highlights how ML models, such as Extra Trees and Random Forest, effectively predict player performance using advanced metrics [9]. Similarly, studies have explored the impact of injuries on team and player performance, identifying correlations between injury types and performance indicators [10]. These works underscore the potential of ML to uncover patterns in complex datasets, such as those found in sports injuries and performance [11]. Drawing from these insights, this study leverages CPET data—a rich source of physiological information—to extend the application of ML to reinjury prediction in soccer players.

In sports, particularly soccer, interest is evolving because of its predictive ability regarding reinjury, especially when athletes recover from heart strain, pulmonary problems, or are in a phase of rehabilitation. The focus on elite soccer players is due to their unique physiological demands and high injury risk, which make CPET data critical for evaluating recovery and reinjury risk. Soccer’s high-intensity activity places significant strain on the cardiovascular and pulmonary systems, making it an ideal model for CPET-based predictions. Additionally, the economic and career stakes in elite soccer justify this targeted approach, which serves as an ideal model for CPET-based predictions while paving the way for broader applications in other sports [12, 13]. Soccer players were chosen as the target population because of the sport’s high physical demands and the significant risk of reinjury following cardiovascular or pulmonary conditions. Soccer requires continuous high-intensity activity, making recovery from such injuries critical for performance and career longevity. Moreover, the physiological stress unique to soccer offers an excellent opportunity to test the effectiveness of CPET-based ML predictions. Yet, despite the raft of information provided by CPET, traditional statistical methodologies often fail to effectively take advantage of this in providing highly specific predictions. Thus, there is a wide gap between available data and actionable insight for the clinician. CPET is a well-established modality for monitoring functional capacity and improvement in a wide range of patient populations, though predicting such specific clinical outcomes as reinjury remains problematic [1, 4]. 

Alternative methods to CPET for reinjury prediction include biomechanical modeling, which examines movement patterns and mechanical stresses to estimate injury risk, and psychological evaluations, which assess mental resilience and recovery preparedness. While these techniques provide valuable information, they lack the quantitative depth of CPET in assessing cardiopulmonary responses [14–16]. 

While machine learning has been applied to CPET data in some studies, the majority of these efforts have focused on diagnostic tasks such as identifying cardiovascular or pulmonary disorders or predicting general exercise tolerance. Studies specifically targeting reinjury prediction are limited and tend to rely on smaller datasets or less advanced methodologies. By contrast, this research uniquely aims to bridge this gap, focusing on the potential of CPET-derived data to predict reinjury, a critical and underexplored area in athlete care [17]. Currently, reinjury prediction in soccer players relies heavily on clinical judgment and basic statistical models, which may not capture the full complexity of an athlete’s physiological response to exercise. This limitation is exacerbated by the lack of comparative studies applying modern machine learning techniques to reinjury prediction in soccer or other sports. Although machine learning has been applied to CPET data in a few studies, these efforts have primarily focused on broad classifications such as disease diagnosis or exercise tolerance rather than specific outcomes like reinjury. There remains a significant gap in leveraging the full predictive power of CPET data to target reinjury, particularly in soccer players recovering from cardiovascular or pulmonary conditions. This gap underscores the need for advanced analytical tools to better interpret the wealth of data CPET provides and translate it into clinically meaningful predictions.

The objective of this study is to develop a machine learning model capable of predicting reinjury in athletes based on CPET data. This research seeks to explore the feasibility and accuracy of using CPET-derived physiological parameters as predictors of reinjury risk. This study applies machine learning algorithms to identify patterns between exercise test variables and reinjury outcomes, which may be missed by traditional methods. Ultimately, the aim is to provide clinicians with a powerful tool that can support decision-making in the prevention of reinjury, enabling more personalized and data-driven patient care.

By identifying complex patterns in the physiological data that are associated with reinjury, the machine learning model could provide clinicians with a more reliable tool for athlete risk stratification. This could lead to earlier interventions and more tailored rehabilitation plans, ultimately reducing the rate of reinjury and improving long-term athlete outcomes.

This research can pave the way for integrating CPET data into clinical decision-making by machine learning predictions. The methodologies demonstrated could extend beyond reinjury prediction to include forecasting other critical outcomes, such as long-term functional capacity or mortality. The growing body of evidence from sports analytics suggests that such ML-driven approaches could significantly enhance the interpretation of physiological data, providing actionable insights for optimizing athlete performance and recovery [9, 11, 18]. As machine learning technology advances, its applications in exercise physiology and healthcare are expected to broaden, presenting significant opportunities to improve athlete care through predictive analytics. Thus, this study not only advances reinjury prediction but also serves as a catalyst for future research and innovations in machine learning and its application to healthcare.

### Related works

Brown et al. (2022) explored the application of neural network methods to enhance the interpretation of Cardiopulmonary Exercise Testing (CPET) data, traditionally analyzed using flowchart-based approaches. Their study rigorously compared the diagnostic performance of flowcharts, principal component analysis (PCA) with logistic regression, autoencoders, and convolutional neural networks (CNN) in diagnosing conditions such as heart failure and metabolic syndrome. The results demonstrated that neural networks, particularly autoencoders, significantly outperformed traditional methods, achieving an impressive 97% accuracy, even with small datasets. These findings underscore the potential of deep learning models to deliver more precise diagnostic assessments in healthcare, especially in scenarios with limited labeled data. However, the authors emphasized the need for further validation with larger datasets and stressed the importance of improving model interpretability to support broader clinical adoption [19]. 

Portella et al. (2022) present a cutting-edge machine learning framework designed to enhance the interpretation of cardiopulmonary exercise testing (CPET) data, addressing the widespread underutilization of CPET in clinical practice. By employing sophisticated feature engineering and selection techniques, particularly through the Boruta algorithm, the researchers developed robust predictive models aimed at identifying primary exercise limitations, including cardiac, pulmonary, and other underlying causes. The study utilized random forest algorithms and leveraged AutoML to optimize the model selection and validation processes while integrating SHAP (SHapley Additive exPlanations) for clear and comprehensive model interpretability. To promote clinical applicability, the authors created an intuitive dashboard featuring insightful visualizations, such as radar plots and summary plots, which enhance decision-making and improve the communication of test results to healthcare professionals. The findings indicate that the cardiac limitation model performed exceptionally well, achieving an impressive area under the curve (AUC) of 0.935. This underscores the transformative potential of machine learning tools to enrich CPET data analysis and improve patient management strategies [20]. 

Shen et al. (2022) explore the utilization of machine learning methodologies, with a particular focus on the XGBoost algorithm, to predict cardiovascular events during exercise evaluations in patients with coronary heart disease (CHD). This study underscores the critical role of feature selection in optimizing model performance, achieving a significant reduction in the number of features from 73 to just 10, while still maintaining a commendable prediction accuracy (AUC = 0.8115). Leveraging data derived from cardiopulmonary exercise testing (CPET), the research identifies vital clinical indicators—including age, gender, diabetes status, and cardiovascular history—that considerably impact the risk of exercise-related events. The results illuminate the potential of machine learning models to deliver real-time risk assessments, thereby enhancing patient safety and guiding effective exercise rehabilitation strategies [21]. 

Huang et al. (2021) explores the efficacy of a Sparse Representation Classifier with Active Learning (SRC-AL) in predicting cardiovascular responses during cardiopulmonary exercise testing (CPET). By leveraging data from three phases of CPET, the research demonstrates that SRC-AL achieves remarkable accuracy rates, such as 1.000 for VO_2_/HR and 0.917 for cardiac output during the exercise phase. Additionally, SRC-AL outperforms traditional baseline methods, showing an overall accuracy of 0.974 on the ECGFiveDays dataset compared to 0.900 for the SRC model and 0.770 for Support Vector Machines (SVM). The F1-scores for various indicators, including 0.737 for VO_2_/HR and 0.720 for cardiac output, reflect the model’s capability to effectively capture the complex dynamics of cardiovascular responses. These findings highlight SRC-AL as a valuable tool for enhancing the assessment of individual responsiveness to aerobic exercise interventions, achieving an accuracy of 0.958 for the recovery phase. Furthermore, the study emphasizes the importance of utilizing machine learning approaches in clinical settings, as they offer superior predictive capabilities compared to conventional methods based on simple metrics like blood pressure changes [22]. 

## Method and material

### Dataset description

The dataset comprises comprehensive records of 256 highly skilled soccer players from the top 16 soccer teams in Iran, collected over an 8-month period in 2023–2024. This dataset offers a rich array of variables to explore, with certain features categorized as binomial, including “History of previous CONCUSSION” and “REInjury,” both of which are recorded as either “Yes” or “No” to indicate the presence or absence of these conditions. The “TEAM” feature is categorical, representing the various soccer teams, and is therefore classified as a polynomial variable. In contrast, all remaining features are numerical and capture a broad spectrum of physiological and performance metrics, providing a detailed profile of each player’s physical and health attributes. Table 1 summarizes the features of this dataset and gives a tabulated overview of the collected variables in a structured manner for further analysis.

**Table 1:** Description of features

No.	Feature	Description
1	Age	Age of the player.
2	Height	Height of the player.
3	Weight	Weight of the player.
4	Team	Soccer team to which the player belongs.
5	CI	Carolina functional performance index, evaluating functional performance and injury risk.
6	Fat	Percentage of body fat.
7	History of previous concussion	Indicates whether the player has a history of previous concussions.
8	VT1	The first ventilatory threshold or anaerobic threshold is, a point where ventilation starts to increase disproportionately, a point where lactate begins to accumulate in the blood (mL/kg/min).
9	VT2	Second ventilatory threshold, is a point where ventilation further increases. It is a higher marker of intensity than VT1. At VT2, lactate has quickly accumulated in the blood and the person needs to breathe heavily. At this rapid rate of breathing, the exerciser can no longer speak.VT2 can also be called the respiratory compensation threshold (RCP).
10	VO_2_peak	The maximal consumption of oxygen achieved during peak exercise (ml kg/min).
11	O_2_puls	Oxygen pulse, the amount of oxygen taken up with each heartbeat. Oxygen pulse (ml/beat) is oxygen uptake (VO_2_) in ml/min divided by heart rate (in bpm).
12	HR1	Threshold heart rate one, corresponding to the first ventilatory threshold.
13	HR2	Second threshold heart rate, corresponding to the second ventilatory threshold.
14	HRmax	Maximum heart rate achieved during exercise.
15	HRlip	Fat-burning heart rate, associated with maximum fat oxidation.
16	VE	Ventilation (VE) is the amount of gas entering and leaving the lungs per minute, and is calculated by multiplying the tidal volume by the respiratory rate.
17	RER	Respiratory exchange ratio, ratio of carbon dioxide output to oxygen intake
18	REInjury	Re-injury occurrence during the season

### Data preprocessing and feature engineering

In the data preprocessing phase, several steps were undertaken to prepare the dataset for machine learning analysis. Categorical variables were converted into numerical format using the LabelEncoder, applied to binary variables such as ‘History of Previous Concussion,’ ‘Team,’ and ‘Reinjury.’ To reduce redundancy, the Body Mass Index (BMI) was calculated using the standard formula, eliminating the need for both weight and height as separate features. Missing values were handled using the SimpleImputer which replaced them with the mean of the respective columns. Alternatively, median imputation could be considered for more robust central tendency measures, if needed for specific variables. Feature scaling was then performed using the StandardScaler, standardizing the numerical features to have a mean of zero and unit variance. This standardization was crucial for ensuring comparability across features, which enhances the performance of machine learning algorithms sensitive to feature magnitudes. Additionally, to capture potential interaction effects between features, Polynomial Features with a degree of 2 were applied. These interaction terms were added to the dataset, and their names were extracted to form a new DataFrame for further analysis.

### Addressing class imbalance

For handling class imbalance within dataset, the Synthetic Minority Over-sampling Technique (SMOTE) was applied [23]. SMOTE synthesizes new samples for the minority class, effectively balancing the class distribution. To mitigate potential bias introduced by SMOTE, we employed cross-validation to assess the model’s performance, ensuring that the synthetic samples did not overly influence the model’s predictions. Additionally, careful consideration was given to imputation, as replacing missing values with the mean could introduce bias. We took care to use the mean (or median where appropriate) of the respective columns to minimize any artificial skew in the data. This step is critical in ensuring that the machine learning model learns effectively from both classes, preventing bias towards the majority class during training.

### Feature selection

Feature selection was performed using Recursive Feature Elimination (RFE) to identify the most relevant predictors for model training [24]. Various machine learning classifiers were utilized as estimators for RFE. The RFE process iteratively removed the least important features based on model performance, continuing until the desired number of features was reached. For this study, 12 features were selected to optimize model accuracy and efficiency in the final.

### Model selection and justification

A diverse set of machine learning algorithms, including both tree-based and non-tree-based methods, was selected to predict re-injury. Each algorithm, optimized for classification tasks, offers distinct advantages, which were compared during the evaluation process.

Extreme Gradient Boosting (XGBoost) is an optimized implementation of gradient boosting that is efficient in both speed and performance [25]. It uses an ensemble of decision trees to minimize errors iteratively, making it highly effective for large datasets and capable of handling missing data [25]. CatBoost is a gradient boosting algorithm that is specifically designed to handle categorical features without the need for extensive preprocessing [26]. It is robust to overfitting and known for its high performance in classification tasks, particularly when dealing with datasets that contain categorical variables [26]. Random Forest is an ensemble learning method that constructs multiple decision trees during training [27]. It combines their outputs to improve classification accuracy and reduce overfitting. Random Forest is known for its robustness and ability to handle large datasets with a high number of features [27]. 

The Decision Tree is arguably the simplest yet most powerful among these models, partitioning data into branches based on feature values [28]. It is equally easily interpretable and visualizable; however, it is prone to overfitting, which can be overcome using ensembling techniques like Random Forest. Support Vector Machine (SVM) is a powerful classification algorithm that works by finding the optimal hyperplane that maximally separates the classes in the feature space [29]. It is particularly effective for high-dimensional data and complex classification problems [29]. Logistic Regression is probably one of the one of the widely used linear model for binary classification [30]. It estimates the probability of a class using a logistic function, making it simple, fast, and highly interpretable for predicting binary outcomes [30]. K-Nearest Neighbors (KNN) is a non-parametric algorithm that classifies instances based on the majority vote of their nearest neighbors in the feature space [31]. Though simple, it is computationally expensive for large datasets, as it requires distance calculation for each instance during prediction.

XGBoost was chosen for its optimized gradient boosting implementation, excelling in speed and performance, particularly with large datasets and missing values. Its ensemble of decision trees iteratively minimizes errors, making it a strong candidate. CatBoost was included for its ability to handle categorical features like ‘Team’ and ‘History of Previous Concussion’ without extensive preprocessing, offering efficiency, resistance to overfitting, and suitability for complex, imbalanced datasets. SVM was selected for its capacity to separate classes in high-dimensional data, capturing non-linear relationships in physiological and performance features through its optimal hyperplane classification. Random Forest was chosen for its robustness in handling large, high-dimensional datasets by constructing multiple decision trees to improve classification accuracy and reduce overfitting. Decision Tree was included for its simplicity and interpretability, enabling visualization of decision-making processes. Its overfitting tendencies were addressed using Random Forest. Logistic Regression was utilized as an efficient, interpretable baseline for binary classification, offering a comparison with more complex models. KNN was included to assess the influence of proximity between instances in feature space, providing insights despite its computational intensity for large datasets.

All models were developed by applying the aforementioned algorithms to each feature set using the Scikit-learn ML library in Python version 3.9.1.

### Hyperparameter tuning with GridSearchCV

The grid search for hyperparameter optimization was done using GridSearchCV. This method had performed an exhaustive search for the best possible hyperparameter configurations across all selected algorithms, using 10-fold cross-validation for the robustness of performance in each model, while finely tuning each model for optimal accuracy and generalization.

### Evaluation metrics

To comprehensively assess model performance, we employed a range of metrics that provide a comprehensive assessment. Accuracy measures the proportion of correctly classified instances, while precision reflects the ratio of true positive predictions to all predicted positives. Recall, also known as sensitivity, indicates the proportion of correctly predicted positive observations among all actual positives. The F1-Score serves as a balanced measure, representing the harmonic mean of precision and recall. We also utilized the Area Under the ROC Curve (AUC) to quantify the model’s ability to differentiate between classes, alongside specificity, which measures the proportion of correctly predicted negative observations. The Matthews Correlation Coefficient (MCC), ranging from − 1 to 1, evaluates the overall quality of binary classifications. Additionally, Log Loss provides insight into the uncertainty of predictions, with lower values preferred, while the Brier Score assesses the accuracy of probabilistic predictions, also favoring lower scores. Finally, Kappa measures the agreement between predicted and actual classifications, correcting for chance agreement. Together, these metrics offer a robust framework for evaluating model performance.

To enhance the reliability of results given the dataset size, cross-validation ensures robust model evaluation, while augmentation strategies like SMOTE and bootstrapping address potential limitations of small datasets by diversifying the sample space. These steps complement the comprehensive evaluation metrics, offering confidence in the generalizability of the findings.

To enhance model interpretability, we also employed SHAP (Shapley Additive Explanations), a powerful tool for explaining individual predictions of machine learning models. SHAP assigns each feature a contribution value to the prediction, enabling us to identify the most important features influencing the model’s decisions. This provides valuable insights into the model’s behavior, improving transparency and trust.

$$\:MCC=\:\frac{\left(TP.TN\right)-(FP.FN)}{\sqrt{(TP+FP)(TP+FN)(TN+FP)(TN+FN)}}\:\:\:\:\:\:\:\:\:\:\:\:\:\:\:\:\:\:\:\:\:\:\:\:\:\:\:\:\:\:\:\:\:\:\:\:\:\:\:\left(1\right)$$

$$\:Log\:Loss=\:-\frac{1}{N}\sum\:_{i=1}^{N}\left[{y}_{i}\text{log}\left({p}_{i}\right)+(1-{y}_{i})\text{log}\left(1-{p}_{i}\right))\right]\:\:\:\:\:\:\:\:\:\:\:\:\:\:\:\:\:\:\:\:\:\:\:\:\:\:\:\:\:\:\:\:\:\:\:\:\:\left(2\right)$$

Where N is the number of observations, $\:{y}_{i}$ is true label (0 or 1) and $\:{p}_{i}$ is predicted probability of the observation being positive.

$$\:Brier\:Score=\frac{1}{N}\sum\:_{i=1}^{N}{({f}_{i}-\:{o}_{i})}^{2}\:\:\:\:\:\:\:\:\:\:\:\:\:\:\:\:\:\:\:\:\:\:\:\:\:\:\:\:\:\:\:\:\:\:\:\:\:\:\:\:\:\:\:\:\:\:\:\:\:\:\:\:\:\:\:\:\:\:\:\:\:\:\:\:\:\:\:\:\:\:\left(3\right)$$

Where $\:{f}_{i}$ is forecast probability for the event, and$\:\:{o}_{i}$ is actual outcome (1 if the event occurs, 0 otherwise)

$$\:Kappa=\:\frac{{P}_{0}-{P}_{e}}{1-{P}_{e}}\:\:\:\:\:\:\:\:\:\:\:\:\:\:\:\:\:\:\:\:\:\:\:\:\:\:\:\:\:\:\:\:\:\:\:\:\:\:\:\:\:\:\:\:\:\:\:\:\:\:\:\:\:\:\:\:\:\:\:\:\:\:\:\:\:\:\:\:\:\:\:\:\:\:\:\:\:\:\:\:\:\:\:\:\:\:\left(4\right)$$

Where $\:{P}_{0}$ is observed agreement (accuracy) and $\:{P}_{e}$ is the expected agreement by chance.

## Results

### Physiological and categorical variables associated with reinjury

The analysis provides valuable insights into the physiological and performance metrics of soccer players, comparing those who experienced reinjury (“YES”) and those who did not (“NO”). Several variables revealed statistically significant differences between the two groups. Age was significantly higher in players with reinjury (Mean: 26.19 ± 5.08) compared to those without (Mean: 24.55 ± 4.52), with a p-value of 0.0359, suggesting that older players may be more prone to reinjury. Similarly, the Carolina functional performance index was higher in the reinjury group (*p* = 0.0136), indicating its potential role as a predictor of injury risk. Heart rate metrics also exhibited notable differences, HRmax was significantly lower in players with reinjury (*p* = 0.0052), indicating diminished peak heart rates. HR2 (submaximal heart rate) was also lower in the reinjury group (*p* = 0.0245). On the other hand, variables such as weight, height, fat percentage (FAT), and VO_2_peak showed no significant differences between the groups, suggesting that these parameters may not be strong predictors of reinjury. A summary of key physiological variables is provided in Table 2.

**Table 2:** Summary of physiological and Performance Metrics for athletes with and without Injury

Feature	Mean ± SD (YES)	Median (YES)	Mean ± SD (NO)	Median (NO)	Mean ± SD	Median	*P*-value
Age	26.19 ± 5.08	26	24.55 ± 4.52	24	24.89 ± 4.66	24	0.0359
Weight	78.2 ± 8.87	76	76.1 ± 7.66	76	76.53 ± 7.95	76	0.1196
Height	182.13 ± 6.69	183	181.48 ± 6.35	182	181.61 ± 6.42	182	0.5239
Carolina functional performance index	33.9 ± 4.16	32.58	32.36 ± 2.93	31.89	32.68 ± 3.28	32.02	0.0136
FAT	11.28 ± 3.02	10.9	11.29 ± 3.16	10.9	11.29 ± 3.13	10.9	0.9694
VT1	30.79 ± 4.33	32	31.99 ± 3.72	32	31.74 ± 3.87	32	0.0678
VT2	50.72 ± 6.59	51	52.04 ± 5.26	52	51.77 ± 5.58	52	0.179
VO_2_peak	54.74 ± 6.79	55	56.17 ± 5.12	57	55.87 ± 5.52	56	0.1568
O_2_puls	24.58 ± 2.84	24	24.12 ± 3.65	24	24.21 ± 3.50	24	0.3203
HR1	131.4 ± 14.2	132	135.25 ± 15.66	136	134.45 ± 15.43	135.5	0.089
HR2	167.62 ± 12.29	168	171.89 ± 11.04	173	171.00 ± 11.42	172	0.0245
HRmax	173.89 ± 11.9	177	178.99 ± 9.56	180	177.93 ± 10.28	179	0.0052
HRlip	119.58 ± 13.1	118	120.33 ± 12.25	119	120.18 ± 12.41	118.5	0.7079
VE	152.6 ± 23.15	149.8	157.12 ± 22.16	157.3	156.18 ± 22.40	156.7	0.2056
RER	1.08 ± 0.07	1.08	1.09 ± 0.06	1.09	1.08 ± 0.06	1.09	0.3215

### Categorical variables and reinjury

In the categorical analysis, a significant association was found between history of previous concussion and reinjury (χ² = 13.0360, *p* = 0.0015), indicating that players with a history of concussion are more likely to experience reinjury. Furthermore, team affiliation was significantly associated with concussion history (χ² = 38.1982, *p* = 0.0174), suggesting that certain teams may have a higher prevalence of concussion incidents. However, team affiliation did not significantly predict reinjury (*p* = 0.5052), indicating that team-based factors alone do not account for reinjury risk (See Table 3).

**Table 3:** Categorical variables and their association with reinjury

Features	χ ^2^	*P*-value
TEAM vs. REInjury	10.2816	0.5052
History of previous CONCUSSION vs. REInjury	13.0360	0.0015
TEAM vs. History of previous CONCUSSION	38.1982	0.0174

The correlation matrix shows the relationships between variables, including those that significantly correlate with reinjury. Age showed weak inverse correlations with VT1, VT2, VO2peak, and HRmax, suggesting that older age is associated with reduced cardiovascular and endurance performance. Height and weight were strongly positively correlated (*r* = 0.81), with taller individuals generally having higher body mass. Height was inversely correlated with cardiovascular measures like VT1 and VT2, indicating that individuals with greater height may have lower endurance thresholds. Weight showed stronger negative correlations with VT2 (*r* = -0.45) and VO2peak (*r* = -0.48), indicating that higher body weight may reduce aerobic capacity and ventilatory thresholds.

The Carolina Index, which measures overall health risk, exhibited weak positive correlations with several variables such as age and fat percentage. However, it had minimal correlation with VO2peak (*r* = -0.16), suggesting that individuals with higher health risks, as measured by this index, may not necessarily have low aerobic fitness levels. Fat percentage demonstrated weak relationships with other variables, but negative correlations with VT1 and VT2 (*r* = -0.17 and *r* = -0.11) suggest that higher fat levels may impair metabolic efficiency and endurance capacity.

A history of previous concussion showed minimal direct correlations with most other variables, though it did correlate weakly with HRmax (*r* = 0.46) and RERM (*r* = 0.48), indicating that concussion history may affect cardiovascular responses during exertion. The strong correlations between VT1, VT2, and VO_2_peak —particularly between VT2 and VO_2_peak (*r* = 0.94)—suggest that individuals who perform well in submaximal exercise tests tend to exhibit better overall aerobic capacity, which is important for predicting reinjury risk associated with physical activity. O_2_puls, reflecting the percentage of oxygen consumption at maximal effort, showed weak but significant correlations with VO_2_peak (*r* = 0.30) and HRmax (*r* = 0.31), suggesting that maximal oxygen uptake is somewhat predictive of cardiovascular performance during exercise. The heart rate metrics (HR1, HR2, and HRmax) showed strong interrelationships, with HRmax being highly correlated with HR1 (*r* = 0.71) and HR2 (*r* = 0.84), emphasizing the role of heart rate as a physiological indicator of reinjury risk, especially during intense physical exertion.

**Fig. 1:**
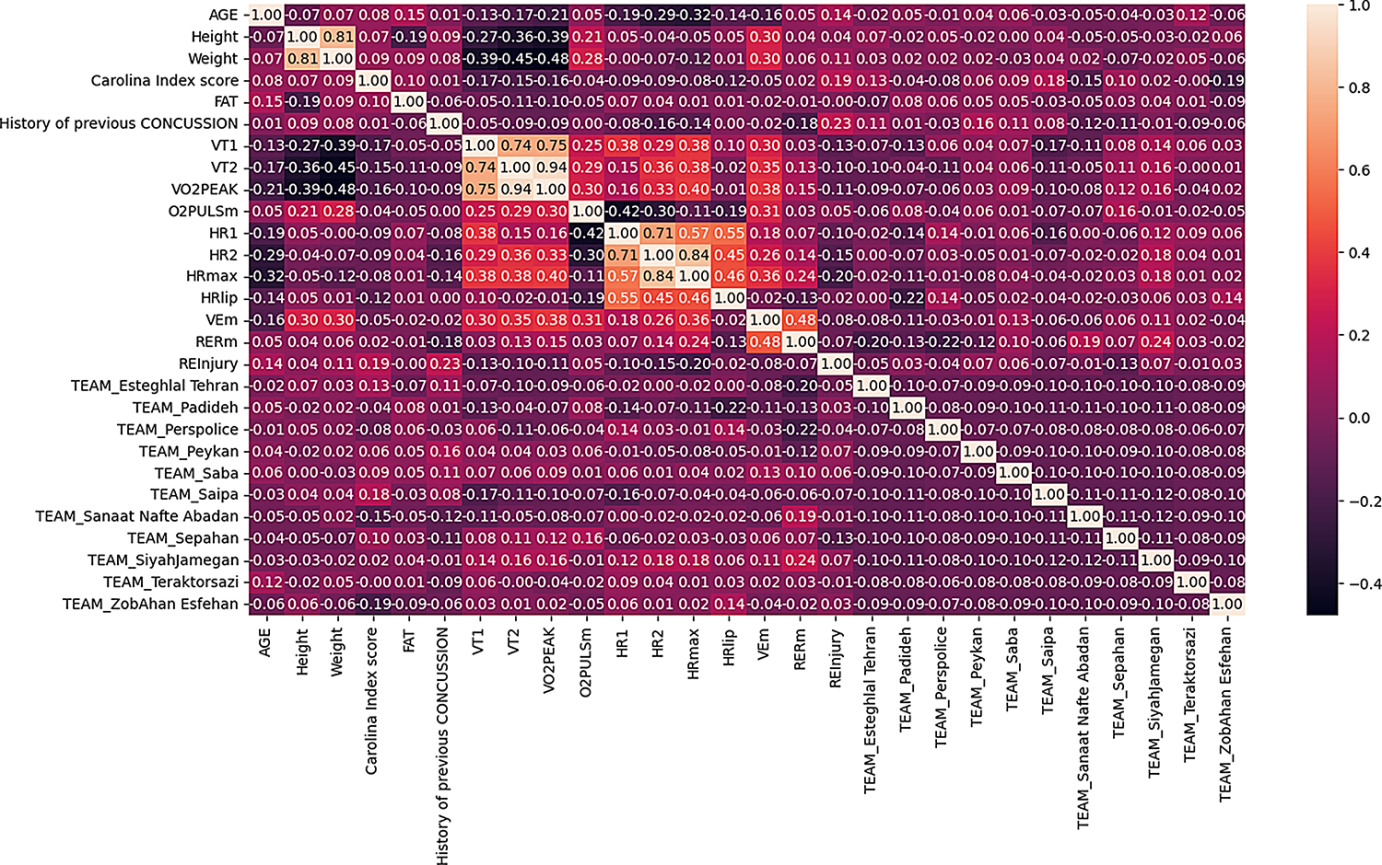
Correlation matrix of variables

The reinjury variable had modest correlations with the Carolina Index (*r* = 0.19), history of previous concussion (*r* = 0.23), and HRmax (*r* = -0.11), suggesting that prior injury history and cardiovascular measures might offer some predictive value for reinjury risk. However, these correlations were relatively weak, indicating that reinjury risk is likely influenced by multiple factors, including biomechanics and psychological aspects, beyond just these physiological metrics. Overall, the analysis highlights the interconnectedness of physical performance measures, with age, body composition, cardiovascular fitness, and injury history playing significant roles in predicting endurance and reinjury risks. These metrics can also be visualized in relation to their correlation, presented in Fig. 1 below.

Table 4 presents a comprehensive overview of the hyperparameter optimization conducted for each machine learning model applied in the study. The tuning process aimed to identify the best hyperparameter settings that maximize model performance in predicting reinjury and ensuring they are well-calibrated for the task of predicting reinjury risk.

**Table 4:** Hyperparameter optimization summary

Model	Hyperparameter	Range of Values	Best Value
**CatBoost**	learning rate	0.01, 0.05, 0.1	0.1
	depth	3, 4, 5, 6, 7, 8	7
	iterations	100, 200, 300	200
	l2_leaf_reg	1, 2, 3, 4, 6	3
**XGBoost**	n_estimators	100, 200, 300	100
	max_depth	10, 15, 20	10
	learning_rate	0.01, 0.05, 0.1	0.1
	min_child_weight	1, 3, 5	3
	subsample	0.7, 0.8, 1.0	0.8
	colsample_bytree	0.7, 0.8, 1.0	0.8
**Random Forest**	n_estimators	100, 200, 300	300
	max_depth	10, 15, 20	10
	min_samples_split	5, 10, 15	5
	min_samples_leaf	2, 4, 6	2
	bootstrap	True, False	True
**SVM**	C	0.1, 1, 10	10
	gamma	‘scale’, ‘auto’	‘scale’
	kernel	‘linear’, ‘rbf’	‘rbf’
**Logistic Regression**	C	0.1, 1, 10	0.1
	penalty	‘l1’, ‘l2’	‘l2’
	solver	‘liblinear’, ‘lbfgs’, ‘newton-cg’	‘liblinear’
**KNN**	n_neighbors	3, 5, 7	7
	p	1 (Manhattan), 2 (Euclidean)	1
	weights	‘uniform’, ‘distance’	‘distance’
**Decision Tree**	criterion	‘gini’, ‘entropy’	‘gini’
	max_depth	None, 10, 20, 30	10
	min_samples_split	2, 5, 10	2
	min_samples_leaf	1, 2, 4, 6	2

Cross-validation techniques were employed to evaluate the models’ performance across different subsets of the data, ensuring that confounders did not unduly affect the generalizability of the results.

### Model performance

A variety of machine learning models were applied to predict reinjury, with CatBoost and SVM achieving the highest performance across several key metrics. CatBoost had the highest accuracy (0.9138), followed closely by SVM (0.9064). In contrast, KNN recorded the lowest accuracy (0.6527), indicating suboptimal performance for this dataset.

CatBoost and SVM demonstrated high precision (0.9038 and 0.8802, respectively) and F1-Scores (0.9148 and 0.9095), indicating a favorable balance between precision and recall. KNN had the highest recall (0.9606), but this came at the cost of lower precision and accuracy, leading to more false positives. Random Forest performed well in terms of precision (0.8929) and specificity (0.8966) but had relatively low recall (0.8621).

In terms of discriminative capability, SVM achieved the highest AUC (0.9725), followed closely by CatBoost (0.9698), indicating excellent ability to differentiate between classes. Logistic Regression, by contrast, recorded the lowest AUC (0.8338), showing reduced effectiveness in class separation.

CatBoost and SVM also achieved the lowest log loss values (0.2182 and 0.2154, respectively), reflecting more confident predictions.

**Fig. 2:**
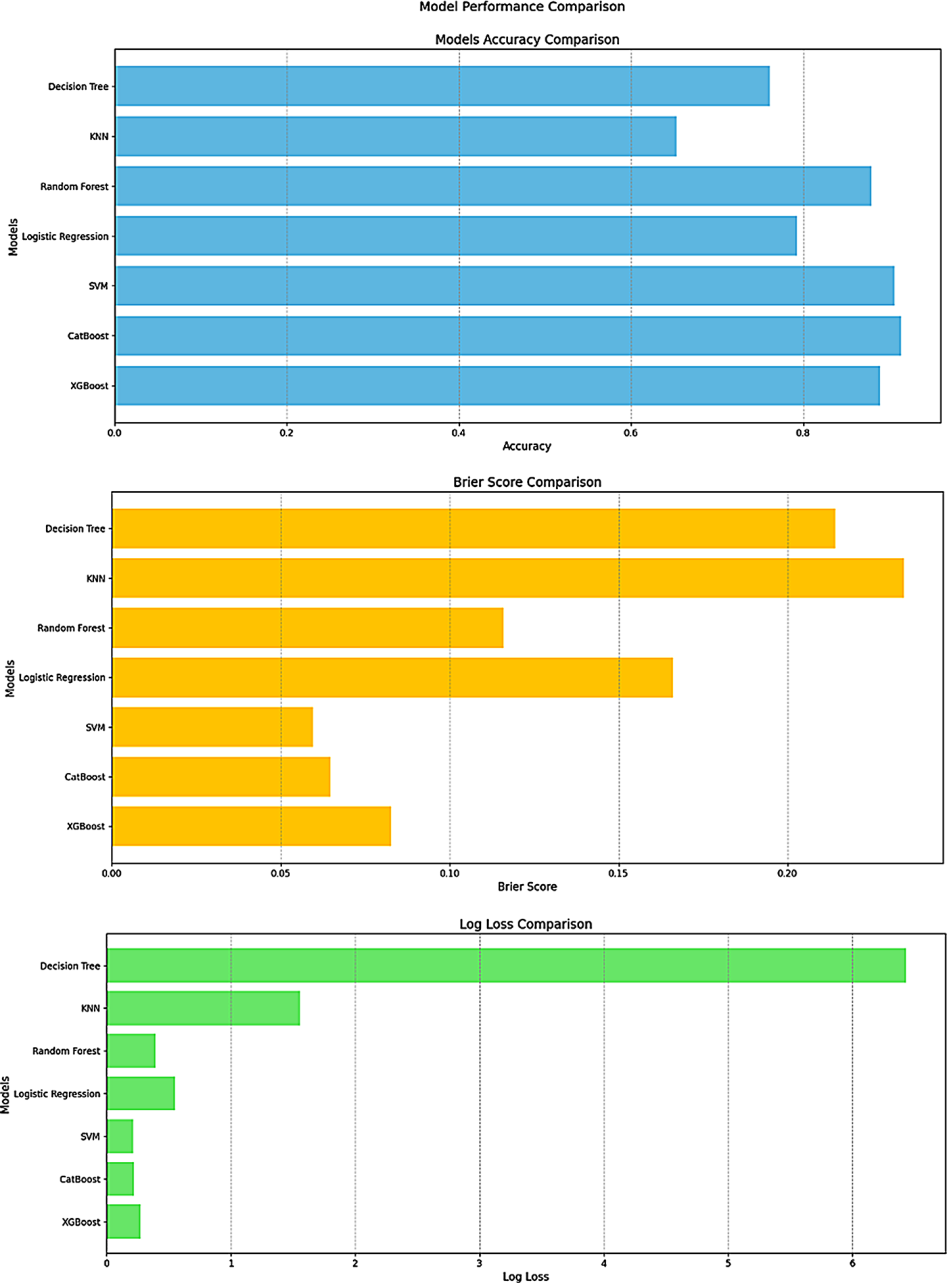
Model performance comparison (accuracy, brier score, and log loss)

The Decision Tree model exhibited particularly poor performance in this regard, with a log loss of 6.4303, indicating substantial uncertainty in its predictions. Additionally, CatBoost and SVM achieved the lowest Brier scores (0.0647 and 0.0595, respectively), indicating superior accuracy in probabilistic predictions. Their Kappa values (0.8276 and 0.8128) further suggest strong agreement between predicted and actual classifications, confirming their reliability as predictive models for reinjury risk. A visual comparison of model accuracy and error metrics is presented in Fig. 2.

Table 5 presents a comprehensive overview of the performance metrics for each machine learning model evaluated, including accuracy, precision, recall, specificity, F1-score, AUC, MCC, log loss, Brier score, and Kappa, providing insights into their predictive capabilities and overall effectiveness in assessing reinjury risk. To ensure robustness and account for the variability in the data, 95% Confidence Intervals (CIs) were computed for each of these metrics using bootstrapping. A bootstrap_ci function was employed to estimate the distribution of each metric, and the 2.5th and 97.5th percentiles were used to calculate the CIs. These intervals provide a clearer picture of the uncertainty in model performance, with all reported metrics now reflecting both the point estimate and the associated confidence range.

KNN has the highest recall (0.9606), meaning it detects most true positives but suffers from low precision (0.5945), leading to many false positives. This may be acceptable in situations where interventions are low-risk. However, the high rate of false positives in KNN could lead to unnecessary treatments and additional patient burden, which could be a significant concern in clinical practice. CatBoost and SVM offer a good balance, with high recall (0.9261 and 0.9409) and decent precision (0.9038 and 0.8802), making them reliable for minimizing both false positives and false negatives. Their high specificity and low false positive rates are critical in avoiding unnecessary medical interventions, while their strong recall ensures that at-risk athletes are flagged appropriately. Random Forest performs well in precision (0.8929) and specificity (0.8966) but has lower recall (0.8621), meaning some reinjury cases might be missed. This could be a problem in clinical scenarios where missing an at-risk patient could result in missed opportunities for treatment. Logistic Regression has a moderate recall (0.8916) and precision (0.7449), making it less effective than CatBoost or SVM in terms of minimizing both false positives and false negatives. Decision Tree and XGBoost show more variability in performance, with higher log loss and lower AUC, indicating less reliability in distinguishing between true positives and negatives.

The choice between models depends on the cost of intervention and the clinical context—if false negatives are more dangerous, models with higher recall, like KNN, might be used, but with caution regarding precision. In clinical applications where false positives could lead to unnecessary procedures, models like CatBoost and SVM, which strike a better balance between recall and precision, would likely be more suitable.

**Table 5:** Model performance metrics with 95% confidence intervals

Model	Accuracy (95% CI)	Precision (95% CI)	Recall (95% CI)	Specificity (95% CI)	F1-Score (95% CI)	AUC (95% CI)	MCC (95% CI)	Log Loss (95% CI)	Brier Score (95% CI)	Kappa (95% CI)
**XGBoost**	0.8892 (0.8571–0.9163)	0.8911 (0.8469–0.9330)	0.8867 (0.8447–0.9282)	0.8916 (0.8916–0.8916)	0.8889 (0.8548–0.9178)	0.9555 (0.9366–0.9736)	0.7783 (0.7192–0.8375)	0.2722 (0.2289–0.3163)	0.0827 (0.0668–0.0999)	0.7783 (0.7177–0.8372)
**CatBoost**	0.9138 (0.8842–0.9409)	0.9038 (0.8660–0.9412)	0.9261 (0.8879–0.9604)	0.9015 (0.9015–0.9015)	0.9148 (0.8829–0.9426)	0.9698 (0.9542–0.9826)	0.8278 (0.7707–0.8814)	0.2182 (0.1681–0.2749)	0.0647 (0.0478–0.0826)	0.8276 (0.7783–0.8803)
**SVM**	0.9064 (0.8768–0.9360)	0.8802 (0.8319–0.9227)	0.9409 (0.9078–0.9709)	0.8719 (0.8719–0.8719)	0.9095 (0.8766–0.9356)	0.9725 (0.9575–0.9856)	0.8147 (0.7575–0.8658)	0.2154 (0.1722–0.2604)	0.0595 (0.0459–0.0763)	0.8128 (0.7539–0.8671)
**Logistic Regression**	0.7931 (0.7562–0.8325)	0.7449 (0.6875–0.7992)	0.8916 (0.8493–0.9324)	0.6946 (0.6946–0.6946)	0.8117 (0.7685–0.8505)	0.8338 (0.7915–0.8718)	0.5979 (0.5188–0.6776)	0.5515 (0.4751–0.6406)	0.1661 (0.1458–0.1883)	0.5862 (0.5063–0.6595)
**Random Forest**	0.8793 (0.8473–0.9113)	0.8929 (0.8502–0.9349)	0.8621 (0.8107–0.9078)	0.8966 (0.8966–0.8966)	0.8772 (0.8416–0.9099)	0.9553 (0.9367–0.9715)	0.7591 (0.6990–0.8180)	0.3923 (0.3684–0.4163)	0.1159 (0.1056–0.1251)	0.7586 (0.6941–0.8173)
**KNN**	0.6527 (0.6059–0.6995)	0.5945 (0.5387–0.6467)	0.9606 (0.9333–0.9848)	0.3448 (0.3448–0.3448)	0.7345 (0.6892–0.7776)	0.8339 (0.7947–0.8723)	0.3876 (0.3146–0.4579)	1.5563 (1.0200–2.1783)	0.2343 (0.2092–0.2618)	0.3054 (0.2315–0.3758)
**Decision Tree**	0.7611 (0.7167–0.8054)	0.7598 (0.7043–0.8159)	0.7635 (0.7044–0.8229)	0.7586 (0.7586–0.7586)	0.7617 (0.7135–0.8051)	0.7885 (0.7455–0.8310)	0.5222 (0.4427–0.6061)	6.4303 (5.1620–7.8064)	0.2141 (0.1801–0.2522)	0.5222 (0.4347–0.6059)

The evaluation of the models based on AUC, MCC, Log Loss, Brier Score, and Cohen’s Kappa provides insights into their performance for clinical applications such as predicting reinjury risk. CatBoost and SVM demonstrated the highest AUC values (0.9698 and 0.9725, respectively), indicating their superior ability to distinguish between athletes at risk and those not at risk of reinjury. These models also achieved high MCC values (0.8278 for CatBoost and 0.8147 for SVM), signifying strong overall performance with a balanced ability to detect reinjury risks while minimizing false positives and false negatives. Both models exhibited low Log Loss (0.2182 for CatBoost and 0.2154 for SVM), reflecting more reliable probability estimates, and achieved excellent Brier Scores (0.0647 for CatBoost and 0.0595 for SVM), highlighting their well-calibrated predictions.

In contrast, KNN and Logistic Regression performed less favorably, with KNN showing a low AUC (0.8339) and a high Log Loss (1.5563), indicating poor calibration and confidence in its predictions. Additionally, KNN (0.3054) and Logistic Regression (0.5862) had lower Kappa values, suggesting less consistent agreement with the actual outcomes. CatBoost and SVM emerged as the most effective models, offering reliable, well-calibrated predictions for clinical decision-making, whereas KNN and Logistic Regression were less reliable in predicting reinjury risk, especially in the context of class imbalance.

**Fig. 3:**
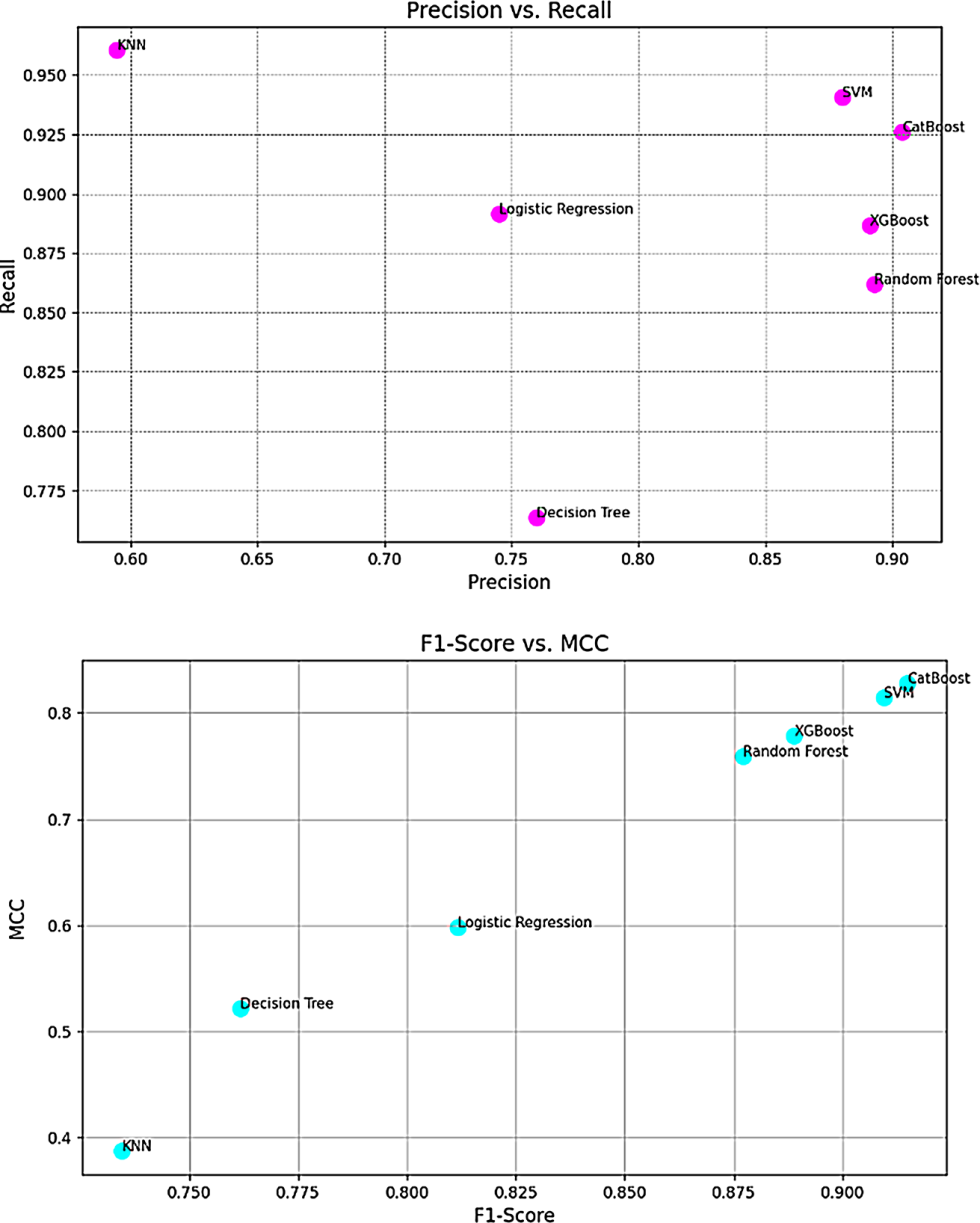
precision vs. recall and F1-score vs. MCC

Figure 3 shows the evaluating of balance between false positives and false negatives, and also relationship between F1-Score and MCC provides further insight into the models’ overall prediction quality.

**Fig. 4:**
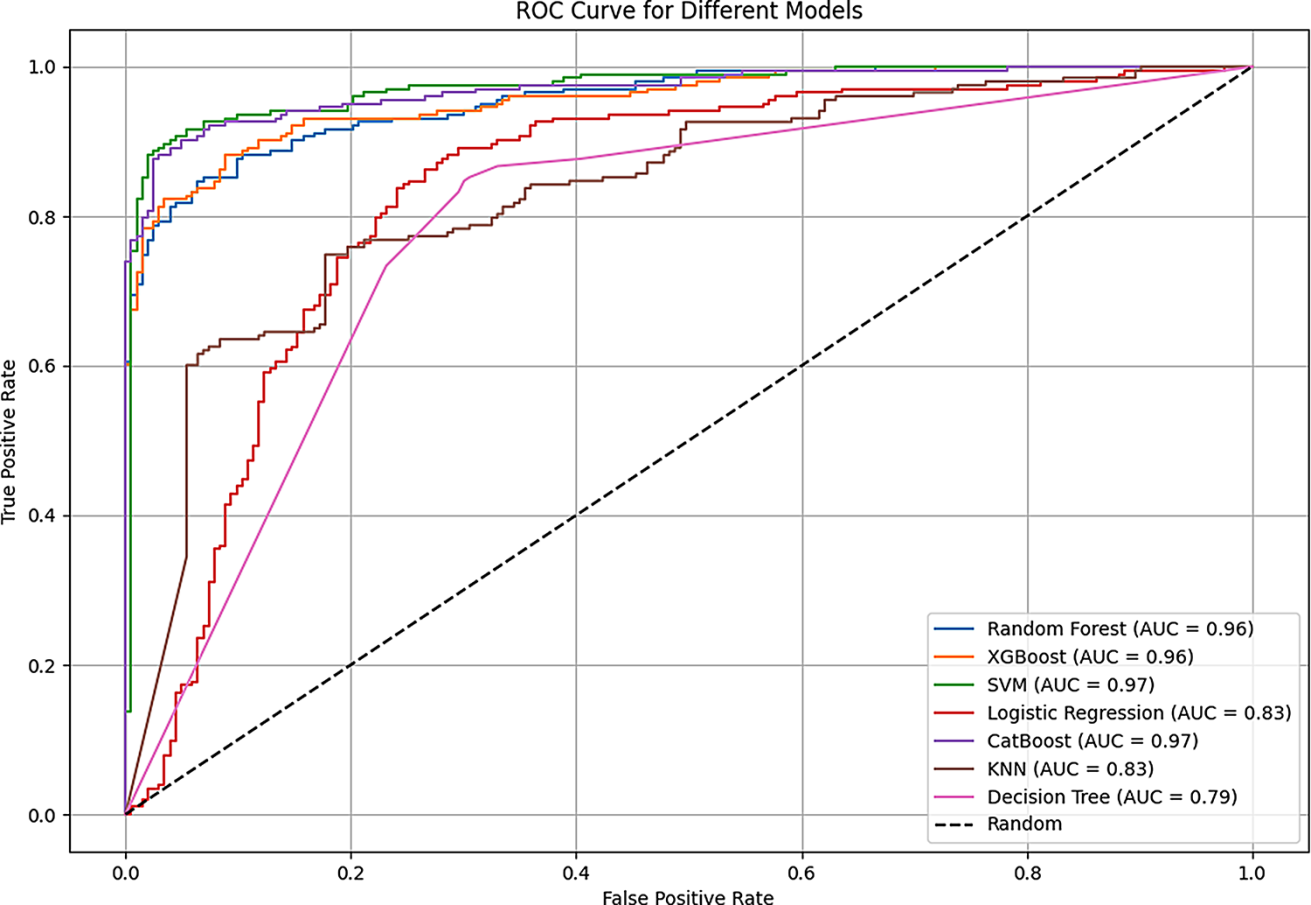
ROC curves for all models

The ROC curves depict the models’ ability to distinguish between the reinjury and non-reinjury groups. To assess the discriminative power of each model, ROC curves were plotted for each classifier in Fig. 4.

**Fig. 5:**
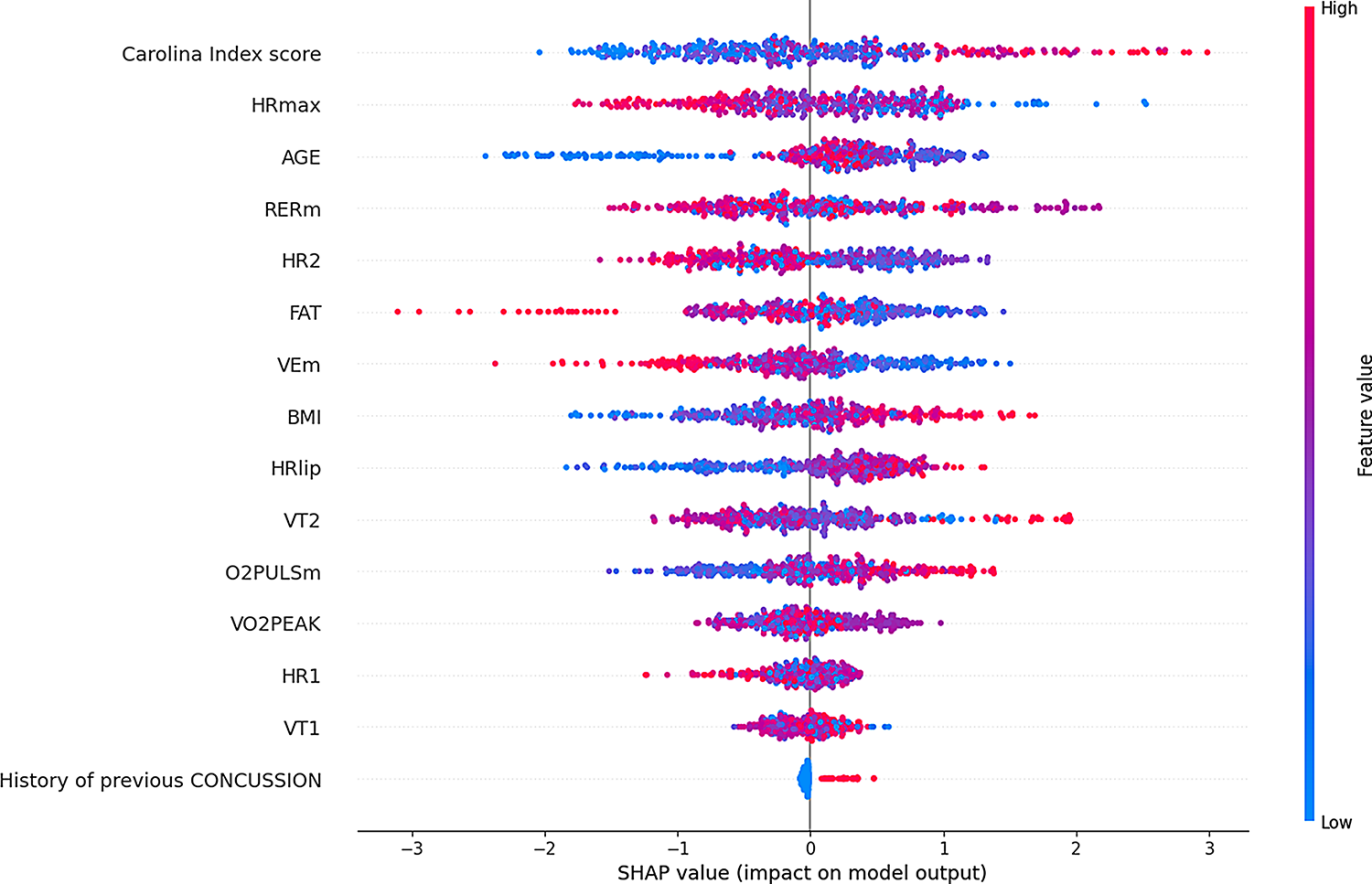
SHAP analysis for CatBoost

**Fig. 6:**
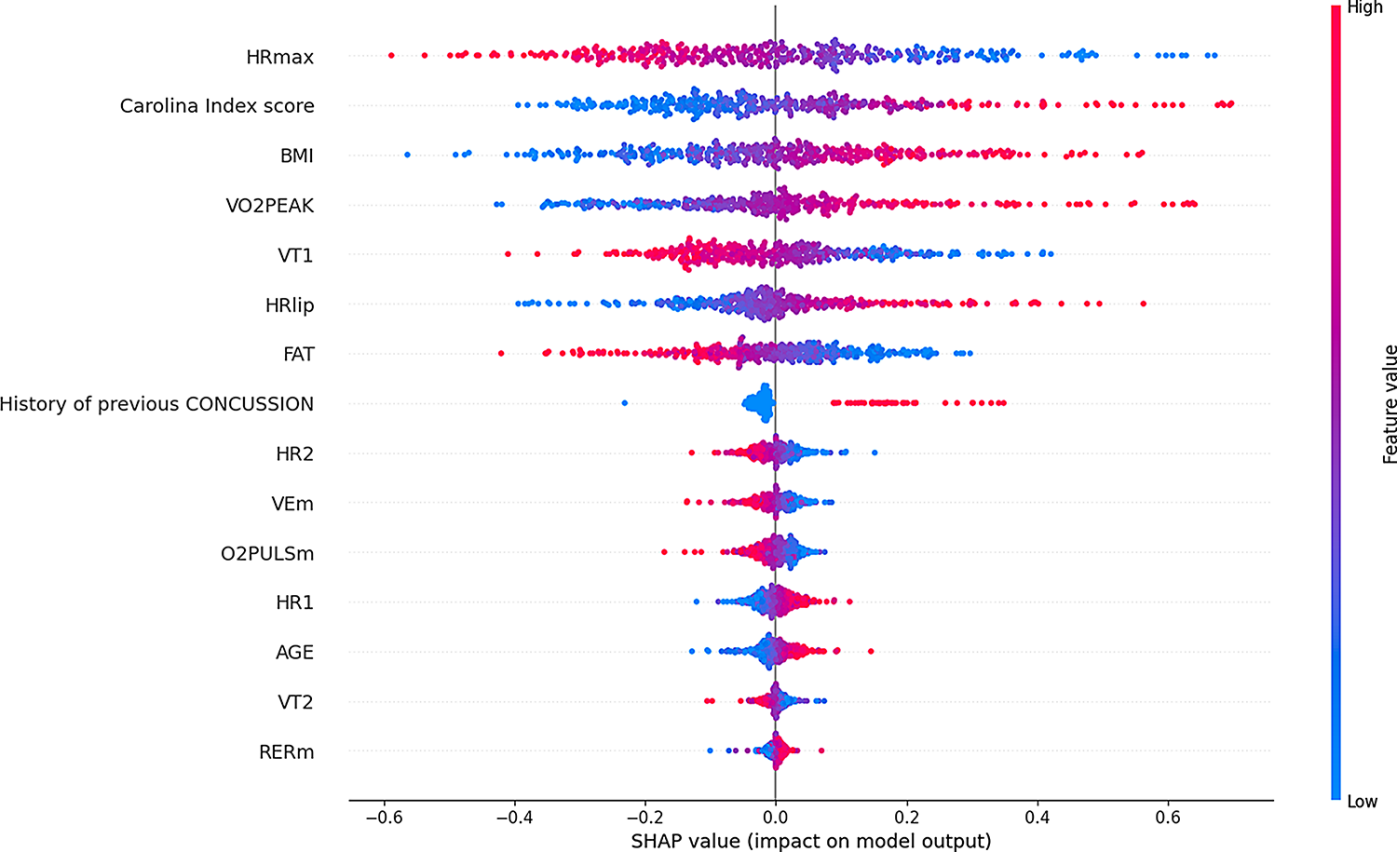
SHAP analysis for SVM

The SHAP analysis identified critical features influencing reinjury risk predictions in the models. For the CatBoost model, the most significant features included the Carolina Index score, HRmax, AGE, RERM, HR2, FAT, BMI, HR1, VT1, VT2, VO2peak, O2pulse, HRlip, VEM, and a history of previous concussions. Figure 5 accentuates these features’ relative importance and directional influence on the model’s outputs, providing clear insights into how parameter variations impact predictions. Similarly, for the SVM model, key features such as HRmax, the Carolina Index score, BMI, VO2peak, VT1, HRlip, FAT, history of previous concussions, HR2, VEM, O2pulse, HR1, AGE, VT2, and RERM were identified. The SHAP values revealed their respective contributions, with feature values ranging from low to high impact on the model output in Fig. 6.

These findings identified the potential of integrating advanced machine learning algorithms with SHAP-based interpretability to improve data-driven clinical decision-making in athlete rehabilitation, enhancing understanding and trust in predictive models.

In a nutshell, while CatBoost and SVM models performed well with excellent AUC values (0.9698 and 0.9725, respectively), variability in predicting accuracy across different subgroups of athletes should be addressed. Age, BMI, and concussion history have varied effects on reinjury risk prediction among these categories. Further evaluation of model performance, especially for athletes with varying concussion histories or cardiovascular fitness levels, could improve model generalizability and personalization. The SHAP analysis identified key features like HRmax, Carolina Index, and BMI as influential in predictions, suggesting that exploring their sensitivity in specific subgroups could provide valuable insights and refine the model’s ability to capture diverse risk factors for reinjury.

### Discussion

In this study, we focused primarily on physiological data derived from CPET, which provided valuable insights into cardiovascular function and endurance capacity. However, factors like psychological readiness and biomechanical conditions—such as movement patterns, muscle imbalances, and joint mechanics—were not included in the analysis. These factors are known to influence injury risk and may interact with the physiological data to affect reinjury outcomes.

The results of this study demonstrate that machine learning models, particularly CatBoost and SVM, offer promising capabilities in predicting reinjury risk among soccer players using CPET data. Both models exhibited high performance across various metrics, including accuracy, precision, recall, F1-score, and AUC, positioning them as valuable tools for clinicians aiming to minimize reinjury risks through more data-driven, personalized rehabilitation protocols.

The findings of this study on CPET metrics, such as HRmax, HR2, VT1, VT2, and VO2peak, highlight their broad applicability across various sports, emphasizing their relevance in injury prevention and performance optimization. CPET is widely recognized as a gold standard for assessing cardiorespiratory fitness, with its metrics offering valuable insights into both endurance capacity and the physiological impact of high-intensity intermittent activities, making it suitable for sports like cycling, running, basketball, and rugby [32]. In addition, CPET metrics can be adapted for different exercise modalities, with studies showing their utility in both treadmill and cycle ergometer settings, which are common in endurance sports [33]. 

CPET data and machine learning models also have been used in healthcare, notably in rehabilitation of patients recovering from cardiovascular diseases, neurological conditions like concussions, or musculoskeletal injuries [21, 34]. Physiological monitoring can guide tailored treatment plans, improving recovery and minimizing reinjury risks. CPET metrics can assess cardiovascular capacity in post-surgical rehabilitation, supporting tailored interventions for better recovery outcomes [21, 34, 35]. 

The findings of this study using CPET data and machine learning models such as CatBoost and SVM, can be applied to high-intensity sports like basketball, rugby, and endurance sports such as running and cycling. CPET metrics, including ventilatory thresholds, VO2max, and HRmax, are valuable for identifying cardiovascular inefficiencies and improving injury prevention strategies [36–38]. 

Real-time monitoring of these metrics using wearable technology introduces transformative possibilities for athlete management. Continuous tracking of metrics such as ventilatory thresholds and VO2max can provide dynamic feedback during training and competition, enabling personalized interventions to optimize recovery and reduce injury risks [39]. Besides, CPET-derived insights into gas exchange and exercise hemodynamics, which differ significantly between trained athletes and the general population, can be utilized to fine-tune training programs and detect potential physiological imbalances [2]. Metrics like VO2max and HRmax, which reflect maximal aerobic and cardiovascular capacity, can be applied to identify overtraining risks and design effective rehabilitation protocols [40]. 

These significant relationships are identified based on the interpretation of the ML models, which analyzed various physiological and biomechanical factors contributing to reinjury risk. The comprehensive analysis indicates the multifactorial nature of injury recurrence in athletes, identifies the interplay between physiological, biomechanical, and historical factors.

A significant association was observed between concussion history and reinjury risk. Aligned with the results of this study, McPherson et al., in their systematic review and meta-analysis, reported that athletes who experienced a concussion had twice the likelihood of suffering a musculoskeletal injury compared to those without a concussion [41]. Athletes with prior concussions are at increased risk of reinjury, likely due to lingering effects on physical performance, balance, and coordination [42]. This emphasizes the need for tailored rehabilitation programs that address both cardiovascular fitness and neuromuscular recovery. Additionally, team affiliation also emerged as a significant factor, potentially reflecting differences in training, medical support, coaching styles, player workloads and recovery protocols across teams [43]. 

The analysis showed no significant relationship between fat percentage in predicting reinjury. Based on the available evidence, body fat percentage mass is associated with the performance of young soccer players [44]. While excess body fat may impair endurance performance, it appears not to directly correlate with injury risk in the same way other variables such as muscle imbalances do. These findings suggested fat percentage alone is not a strong predictor of injury, especially in athletes with adequate cardiovascular conditioning.

The analysis highlighted a notable association between HR2 and history of previous concussion, which showed a strong relationship with reinjury. The meta-analysis by Wesolowski et al. demonstrated that individuals with a history of concussion may exhibit reduced HRV values that persist even after symptomatic recovery [45]. Athletes with a history of concussion displayed lower HR2 values, indicating that their cardiovascular response to exercise was compromised, particularly at higher intensities. The HR2 is a critical marker of an athlete’s capacity to sustain high-intensity efforts before experiencing significant fatigue. A lower HR2 suggests diminished aerobic efficiency and impaired physiological response during high-stress physical activity. This reduction in HR2 could be a lingering effect of prior concussions, which are known to affect not only neurological function but also cardiovascular regulation and autonomic control [46]. The body’s ability to efficiently manage increased workloads may be compromised after concussion, leading to greater fatigue, slower reaction times, and a heightened risk of reinjury. The strong relationship between HR2 and reinjury risk in athletes with a history of concussion further emphasizes the need for tailored rehabilitation programs that address both cardiovascular conditioning and neuromuscular recovery [47]. 

Moreover, a strong correlation between VT1 and HR1 was found. This relationship reflects shared physiological significance, where lower VT1 values correspond to reduced aerobic capacity. Athletes with a lower VT1 often reach the first ventilatory threshold at lower heart rates, reflecting a reduced aerobic capacity. This suggests that the individual’s cardiovascular system struggles to maintain a steady supply of oxygen to muscles during moderate-intensity exercise, which could predispose them to fatigue earlier, increasing the risk of poor performance or reinjury [48]. 

The analysis revealed a significant relationship between body fat percentage and VT2, which represents the point where ventilation increases disproportionately to oxygen consumption, marking the shift to anaerobic metabolism. Fiana et al.‘s study also reveals a significant correlation between body fat percentage and VO_2_max [49]. Athletes with higher body fat percentages exhibited lower VT2 values, meaning they reached their anaerobic threshold earlier than leaner athletes. This suggests that higher body fat negatively impacts cardiovascular efficiency, limiting the ability to sustain prolonged aerobic work. Excess fat acts as both a mechanical and metabolic burden, increasing the energy cost of movement and reducing oxygen delivery to muscles [50]. This reduced capacity to maintain aerobic activity beyond the second ventilatory threshold contributes to early fatigue, limiting performance and potentially increasing the risk of reinjury.

A notable relationship was observed between a history of concussion and elevated BMI (calculated by authors). Unlike the findings of our study, Bramley et al. did not identify a statistically significant relationship between BMI and football-related concussions [51]. It seems the athletes with a history of concussions tended to have higher BMI values, suggesting that individuals who have experienced concussions might struggle with maintaining optimal body composition post-injury. This could be due to prolonged recovery periods following a concussion, leading to reduced physical activity, weight gain, or changes in metabolism. Bramley et al. also reported that, after a concussion, overweight and obese individuals are more likely to exhibit irritability and neurologic disorders [51]. Higher BMI, in conjunction with the residual neurological effects of concussion, may exacerbate the risk of reinjury. Increased body weight can lead to additional stress on joints and muscles during physical activity, potentially contributing to movement inefficiencies or biomechanical imbalances. When combined with the potential cognitive and motor deficits following concussion, this could explain the heightened reinjury risk observed in athletes with both higher BMI and a history of concussion.

One notable finding of this research was the significant correlation between the Carolina functional performance index, which measures functional performance, and a history of concussions. Athletes with higher Carolina functional performance index were more likely to have experienced multiple concussions. This finding suggests that repeated concussions may lead to long-term functional deficits, delayed reaction times, and compromised motor coordination. These deficits may increase the likelihood of reinjury, particularly in scenarios requiring quick decision-making and precise motor control [52]. 

The relationship between HR2 and RER provides insight into cardiovascular efficiency and metabolic function. HR2, the second threshold heart rate, marks the transition to anaerobic metabolism, while RER reflects the ratio of carbon dioxide output to oxygen uptake, signaling reliance on anaerobic energy pathways. Athletes with higher HR2 values showed greater cardiovascular efficiency, maintaining aerobic metabolism longer. Simultaneously, lower RER values suggest a reliance on aerobic processes, improving endurance. This connection highlights the importance of delayed anaerobic onset and efficient energy utilization for reducing reinjury risk during high-intensity exercise [53]. 

The strong correlation between VO_2_peak, a measure of maximal oxygen uptake during peak exercise, and HRmax, the highest heart rate during exertion, accentuates the combined significance of maximal oxygen uptake and peak cardiovascular output in injury prevention. Athletes with higher VO_2_peak values tend to exhibit higher HRmax, reflecting superior cardiovascular and respiratory systems capable of sustaining high-intensity efforts. This relationship suggests that maintaining optimal aerobic capacity is critical for tolerating the physical demands of sport, thereby reducing the strain on the body and lowering the risk of reinjury. Our results align with the findings of Watson et al., who reported that early-season injuries in soccer players are associated with aerobic fitness and lean body mass [54]. 

O_2_puls, representing the amount of oxygen consumed per heartbeat at peak exertion, showed a significant decline with age. This aligns with existing literature indicating that advancing age compromises cardiovascular efficiency due to reduced cardiac output and muscle mass [55]. Lower O_2_puls values in older athletes may limit their ability to transport and utilize oxygen effectively, thereby increasing the risk of fatigue, compromised performance, and injury. These age-related physiological changes necessitate tailored conditioning programs that account for declining aerobic capacity in older athletes.

A significant relationship between the Carolina functional performance index and O_2_puls highlights the role of cardiovascular fitness in reducing reinjury risk. Athletes with higher Carolina functional performance index, indicating better overall fitness, exhibited more efficient oxygen utilization during peak exertion. This suggests that enhanced cardiovascular efficiency may serve as a protective factor against reinjury, as athletes with higher O_2_puls values are better equipped to meet the physical demands of sport, reducing the likelihood of injury recurrence.

HR1, the heart rate at which athletes transition from rest to moderate exercise, was found to increase with age. This aligns with research showing that aging impacts cardiovascular function, requiring older athletes to exert more effort to reach aerobic thresholds. The higher HR1 values in older athletes suggest a diminished aerobic capacity, which can lead to increased fatigue during training and competition, heightening injury risk. Age-specific conditioning strategies that address these changes in cardiovascular efficiency may help mitigate this risk. This is consistent with the findings of Malone et al., who suggested that a higher intermittent aerobic capacity seems to provide better injury protection when elite soccer players experience sudden increases in workload [56]. 

The positive correlation between O_2_puls and HRlip, fat-burning heart rate, indicates that athletes who efficiently use oxygen during maximal exertion are able to reach higher heart rates before significant lactate accumulation. This finding reinforces the importance of aerobic fitness in sustaining high-intensity performance and delaying the onset of fatigue [57], both of which are essential in reducing reinjury risk during prolonged or strenuous activities.

Finally, the significant relationship between the Carolina Index and BMI suggests that higher BMI values are associated with a less favorable injury risk profile. Elevated BMI can place greater stress on musculoskeletal structures, reduce agility, and impair biomechanical stability, all of which can contribute to a higher likelihood of injury. The findings of Alangari et al. have also shown that a higher BMI is linked to an elevated risk of musculoskeletal injuries [58]. This underscores the need for targeted conditioning and body composition monitoring to optimize athletic performance and minimize reinjury risk in athletes with elevated BMI.

CPET data and machine learning models also have applications in healthcare, particularly in rehabilitation for patients recovering from cardiovascular diseases, neurological conditions like concussions, or musculoskeletal injuries. Physiological monitoring can guide personalized treatment plans, improving recovery and minimizing reinjury risks. CPET metrics can assess cardiovascular capacity in post-surgical rehabilitation, supporting tailored interventions for better recovery outcomes.

### Limitations

This study focused on elite soccer players from Iran, which may limit the generalizability of its findings to other sports or demographics. The relatively small sample size (*n* = 256) further restricts the model’s applicability to larger and more diverse athletic populations. Expanding future research to include data from different sports and geographical regions would enhance the robustness and versatility of the developed models, broadening their range of applications.

Although CPET data provides valuable physiological insights, the study did not account for external factors such as psychological readiness, training intensity, and biomechanical conditions, all of which could interact with CPET-derived metrics and influence reinjury risk. For instance, psychological readiness significantly impacts recovery and performance, potentially affecting how the body responds to physical stress. Similarly, biomechanical factors, such as movement patterns and muscle imbalances, directly contribute to injury susceptibility.

Another critical consideration is the representativeness of the dataset. The sample is limited to a specific population of elite soccer players from Iran, which may not reflect the broader athletic population, particularly in other regions or sports. This limitation could affect the model’s generalizability across diverse demographics. The relatively small sample size also increases the risk of overfitting, especially in complex models like CatBoost, which can perform well on the training set but may fail to generalize to unseen data. To mitigate this, future research should include larger, more diverse datasets to reduce the risk of overfitting and improve model reliability.

Another critical consideration is the scalability of these models for real-time, team-wide applications. While the study demonstrates strong individual prediction capabilities, real-world implementation faces challenges such as data collection logistics, model updates, and integration with existing systems. Future research should address these challenges by exploring the feasibility of real-time data integration and investigating the incorporation of these models into training and recovery platforms to support immediate decision-making for athletes, coaches, and medical staff.

Finally, further validation on external datasets is essential to establish a more reliable and generalized model, ensuring that the findings are applicable across various contexts and populations.

## Conclusion

This study aimed to explore the utility of machine learning models, specifically CatBoost and Support Vector Machine (SVM), in predicting reinjury risk among athletes by leveraging Cardiopulmonary Exercise Testing (CPET) data. The primary objective was to identify key physiological and historical factors, such as heart rate metrics and concussion history, that contribute to reinjury risk in athletes. Our findings demonstrate that these models provide a more robust and precise method for assessing injury risk compared to traditional clinical approaches. By incorporating CPET-derived data into predictive models, clinicians are equipped with a powerful tool to develop individualized rehabilitation protocols, which can play a critical role in mitigating reinjury rates and optimizing athletic recovery. This research highlights the increasing significance of machine learning techniques in sports medicine, underscoring their potential to enhance injury prevention strategies and improve long-term athlete outcomes.

### Future works

Building on this study, future research should focus on expanding the dataset to include a larger and more diverse sample from various sports and populations, which would improve the generalizability of the model. This expansion should also incorporate key external factors, such as training load, psychological readiness, and biomechanical metrics, to create a more holistic and accurate system for predicting reinjury risk. Additionally, external validation on independent datasets is crucial to ensure the robustness and reliability of the predictive models across different contexts and athlete demographics. Cross-dataset comparisons will strengthen the model’s applicability and help mitigate concerns about overfitting, ensuring that the findings can be generalized beyond the current dataset.

A critical next step is the integration of these models with wearable technologies, which could allow for continuous real-time monitoring and dynamic risk assessments. By embedding the models into devices that track physiological data (e.g., heart rate, movement patterns, fatigue levels), it would be possible to generate timely insights for clinicians, coaches, and athletes during training and competition. This real-time feedback could support more proactive, personalized rehabilitation protocols and injury prevention strategies, leading to a more efficient and individualized approach to athlete care. Future work should focus on optimizing data collection methods, ensuring that they are non-intrusive, accurate, and compatible with wearable technologies commonly used in sports.

Moreover, the development of automated systems for data processing and model application will be essential for practical implementation. Such systems should aim to streamline data collection, minimize human error, and provide actionable insights in a user-friendly format, making them accessible for both clinical and sports settings. Research could also investigate how these models can be integrated with sports management software or injury tracking systems for team-wide applications, offering scalable solutions for injury prevention at both the individual and team levels.

This could lead to the developing of app-based solutions or team-wide monitoring systems that could track and manage reinjury risk in real-time, facilitating better-informed decisions in sports teams and clinical rehabilitation settings.

Finally, future studies should explore how the machine learning models can be adapted for use in other health contexts, such as evaluating exercise tolerance in cardiac patients or predicting long-term recovery outcomes, broadening their impact across healthcare domains.

## Data Availability

The datasets and code used and/or analyzed during this study are available from the corresponding author upon reasonable request.
